# Post‐Tuberculosis Adrenal Crisis in a Young Boy: A Case Report

**DOI:** 10.1002/ccr3.71345

**Published:** 2025-10-24

**Authors:** Aisha Haleem, Saad Ashraf, Daniya Tarique, Muhammad Zohair, Ajeet Singh, Biruk Demisse Ayalew

**Affiliations:** ^1^ Department of Pediatrics Ruth K. M. Pfau, Civil Hospital Karachi (CHK), Dow University of Health Sciences Karachi Pakistan; ^2^ Department of Medicine Dow University of Health Sciences Karachi Pakistan; ^3^ Department of Nephrology Sindh Institute of Urology and Transplantation (SIUT) Karachi Pakistan; ^4^ St. Paul's Hospital Millennium Medical College Addis Ababa Ethiopia

**Keywords:** adrenal crisis, hyperpigmentation, primary adrenal insufficiency, tuberculosis

## Abstract

An adrenal crisis should be considered in children with a history of tuberculosis who present with shock, hypoglycemia, and hyperpigmentation. Even years after successful treatment, TB can cause irreversible adrenal damage leading to primary adrenal insufficiency. Early recognition and prompt glucocorticoid replacement are lifesaving. Routine screening in high‐risk patients may prevent delayed diagnosis and fatal outcomes.

**Abbreviations**
ACTH: adrenocorticotropic hormone
ATT: anti‐tuberculous therapy
CT: computed tomography
HPA: hypothalamic–pituitary–adrenal
IV: intravenous
MRI: magnetic resonance imaging
PAI: primary adrenal insufficiency
TB: tuberculosis

## 1 Introduction

Tuberculosis (TB) primarily affects the lungs but commonly disseminates to extrapulmonary sites, including the adrenal glands, particularly in endemic settings. While autoimmune adrenalitis is the leading cause of primary adrenal insufficiency (PAI) in high‐income countries, tuberculosis is a leading cause of adrenal insufficiency in developing countries, accounting for 7%–20% of primary adrenal insufficiency cases. The adrenal glands consist of the cortex, which produces aldosterone (zona glomerulosa), cortisol (zona fasciculata), androgens (zona reticularis), and the medulla, which secretes catecholamines. Adrenal insufficiency results from inadequate production of adrenal cortical hormones and is classified into three types: primary, secondary, and tertiary. Primary adrenal insufficiency arises from direct adrenal gland damage due to causes such as autoimmune diseases [1, 2], infections (e.g., TB, HIV, cytomegalovirus), hemorrhage, trauma, or infiltrative disorders like hemochromatosis or sarcoidosis [3, 4]. Secondary adrenal insufficiency often results from exogenous glucocorticoid use, suppressing the hypothalamic–pituitary–adrenal (HPA) axis. Tertiary adrenal insufficiency is caused by hypothalamic or pituitary dysfunction, such as from tumors, trauma, or congenital deficiencies [5].

Adrenal involvement typically occurs via hematogenous spread and may manifest years after the initial infection, complicating diagnosis. This case report describes a pediatric patient with adrenal crisis secondary to prior pulmonary tuberculosis (TB), highlighting the need for increased awareness to facilitate early diagnosis and improved outcomes.

## 2 Case Presentation

An 8‐year‐old unvaccinated male with no documented history of BCG vaccination and no visible BCG scar presented to our emergency department with a 1‐day history of fever, non‐bilious vomiting, and watery diarrhea, accompanied by decreased oral intake and progressive lethargy. His medical history included sputum‐positive pulmonary TB diagnosed at age 2, treated with 6 months of anti‐tuberculous therapy (ATT). Over the preceding 6 years, he had multiple hospital visits for lethargy and hypoglycemia, misdiagnosed as sepsis, but he never received a definitive diagnosis.

**FIGURE 1:**
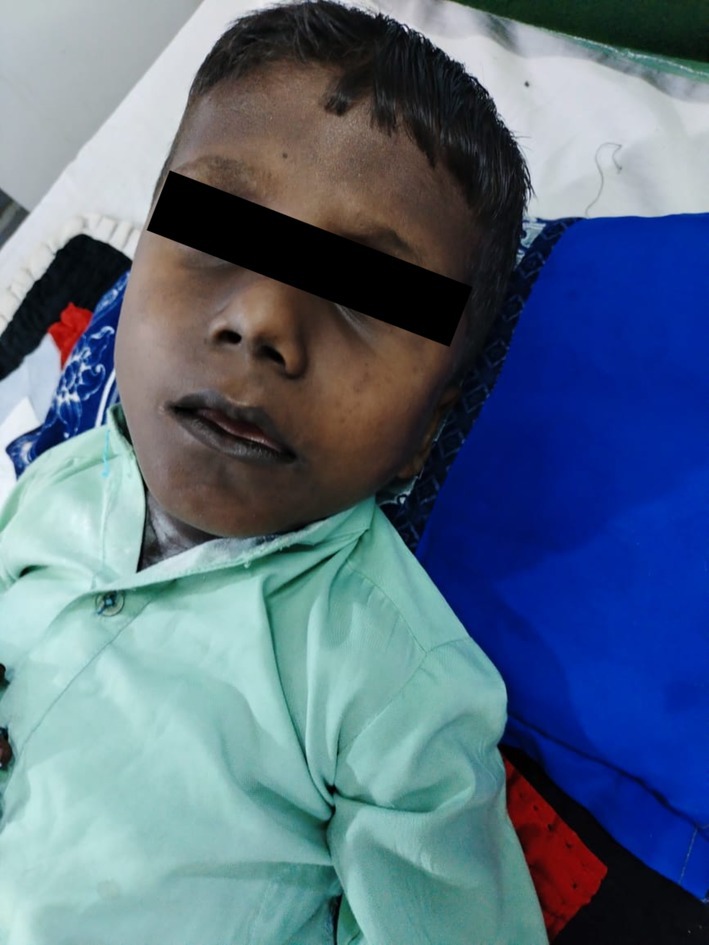
Generalized hyperpigmentation involving the face and trunk noted on presentation. This distribution is consistent with primary adrenal insufficiency.

**FIGURE 2:**
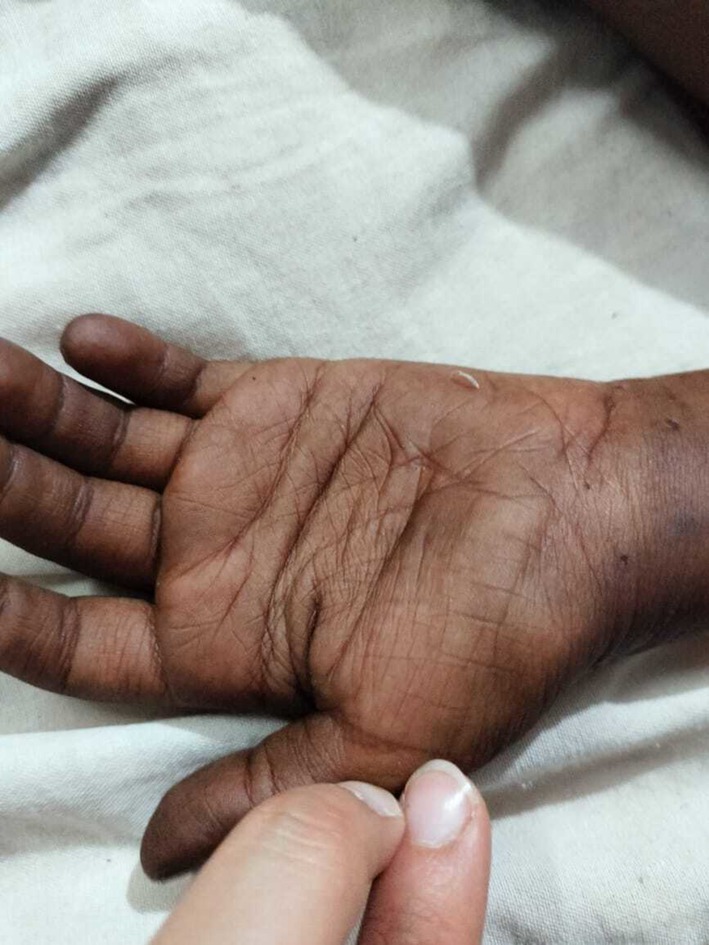
Hyperpigmentation of palmar creases, photographed on presentation. The accentuated palmar creases are characteristic of chronic hyperpigmentation from elevated ACTH.

On arrival, the patient was hypovolemic and hypotensive (blood pressure 50/25 mmHg) and experienced a generalized tonic–clonic seizure associated with hypoglycemia (blood glucose 36 mg/dL). Physical examination revealed generalized skin hyperpigmentation, darkened gums, and accentuated palmar creases (Figures 1 and 2). The patient had no prior surgeries, regular medications, or known drug allergies. There was no family history of adrenal disease, early deaths, or tuberculosis, and parental consanguinity was negative. Systemic review was negative for chronic cough, weight loss, polyuria/polydipsia, dysphagia, alacrima, autonomic dysfunction, meningeal signs, skin infections, or developmental regression.

## 3 Differential Diagnosis, Investigations, and Management

Initial management included 10% dextrose water and normal saline boluses to address hypoglycemia and hypovolemia. Laboratory tests before fluid resuscitation showed low early morning serum cortisol (0.29 μg/dL, reference: 6–23 μg/dL), elevated ACTH (1250 pg/mL, reference: 10–50 pg/mL), and normal electrolytes (sodium 138 mmol/L, potassium 4.2 mmol/L) (Table 1). A contrast‐enhanced abdominal CT revealed bilateral adrenal atrophy, consistent with chronic adrenal damage. High‐resolution computed tomography (HRCT) of the chest showed left segmental fibrosis, likely a residual effect of prior TB. Brain MRI and very long‐chain fatty acid (VLCFA) levels were within normal limits, effectively excluding X‐linked adrenoleukodystrophy. Allgrove syndrome was also considered unlikely, as the patient exhibited no features of autonomic dysfunction, achalasia, or alacrimia. Lumbar puncture was deferred due to a normal neurological examination and absence of meningeal signs.

**TABLE 1:** Laboratory and imaging findings.

Parameter	Result	Reference range	Comments
Morning serum cortisol (8 a.m.)	0.29 μg/dL	4.82–19.5 μg/dL	Low level indicative of adrenal insufficiency
Morning plasma ACTH (8 a.m.)	> 1250 pg/mL	10–50 pg/mL	Markedly elevated; diagnostic of primary insufficiency
Sodium	138 mmol/L	135–145 mmol/L	Normal
Potassium	4.2 mmol/L	3.5–5.0 mmol/L	Normal
Blood glucose	36 mg/dL	70–110 mg/dL	Hypoglycemia
Abdominal CT	Bilateral adrenal atrophy	Normal adrenal glands	Consistent with chronic damage
Chest HRCT	Left segmental fibrosis	Normal lung parenchyma	Likely TB sequelae
Brain MRI	Normal	Normal	Rules out adrenoleukodystrophy
VLCFA	Normal	Normal	Rules out adrenoleukodystrophy

The patient received stress‐dose intravenous hydrocortisone (100 mg/m^2^/day) and was transitioned to maintenance oral hydrocortisone (15 mg/m^2^/day) and fludrocortisone (0.1 mg/day) (Table 2). A sick‐day protocol, involving doubling glucocorticoid doses during illness or stress, was provided to the family. Two hours after treatment initiation, the patient regained consciousness with no neurological deficits.

**TABLE 2:** Management protocol.

Intervention	Dose/Description
Initial fluid resuscitation	10% Dextrose + normal saline boluses
Hydrocortisone (stress dose)	100 mg/m^2^/day IV
Hydrocortisone (maintenance)	15 mg/m^2^/day oral
Fludrocortisone	0.1 mg/day oral
Sick‐day protocol	Double glucocorticoid dose during illness/stress

## 4 Outcome and Follow‐Up

At a 1‐month follow‐up, the patient was stable, with improved energy levels and no further episodes of hypoglycemia or hypotension. Morning (08:00) serum cortisol was 12.5 μg/dL (reference: 6–23 μg/dL), and ACTH was 45 pg/mL (reference: 10–50 pg/mL), indicating adequate glucocorticoid replacement. Electrolytes at one month showed sodium 140 mmol/L (reference: 135–145 mmol/L) and potassium 4.0 mmol/L (reference: 3.5–5.0 mmol/L). Growth parameters at one month included weight at the 25th percentile (22 kg) and height at the 30th percentile (122 cm) for age. The patient continued maintenance therapy with hydrocortisone (12 mg/m^2^/day) and fludrocortisone (0.1 mg/day), and adhered to the sick‐day protocol.

## 5 Discussion

This case highlights a classic presentation of primary adrenal insufficiency (Addison disease) that progressed to an adrenal crisis, which is a life‐threatening emergency in a pediatric patient with a prior history of pulmonary tuberculosis (TB). Primary adrenal insufficiency is a rare entity, with an estimated prevalence of 100–140 per million in developed countries [6]. It is less common in children and often presents with nonspecific symptoms such as fatigue, weight loss, and hyperpigmentation, leading to diagnostic delays [7, 8]. Tuberculosis has reportedly been a major cause of Addison's disease and remains the leading infectious etiology of adrenal insufficiency in underdeveloped and developing countries [9]. The ability of TB to disseminate hematogenously coupled with the adrenal glands' rich blood supply makes these organs vulnerable to be affected [10]. The clinical signs may take years to develop, or the infection may remain completely asymptomatic, making it difficult to achieve a diagnosis.

This patient was diagnosed with pulmonary TB at the age of 2, for which he received 6 months of anti‐tuberculous therapy (ATT). He was kept under close follow‐up; during this time period, his growth parameters were observed. After ATT completion, Gene Xpert was done (from gastric lavage) and it was negative. He had on‐and‐off admission to the hospital due to lethargy and hypoglycemia; however, no definitive diagnosis was made. In his current admission, he presented with hypotensive shock, hypoglycemia, and hyperpigmentation, combined with low cortisol, elevated ACTH, and bilateral adrenal atrophy indicating primary adrenal insufficiency secondary to prior TB. The normal electrolyte profile was unexpected, as hyponatremia and hyperkalemia are common in adrenal crisis. The absence of findings suggestive of Allgrove syndrome or adrenoleukodystrophy further supported the diagnosis of post‐TB adrenal insufficiency.

Managing Addison's disease alongside tuberculosis is particularly challenging because rifampicin, which is a key antitubercular drug, is a strong inducer of the cytochrome P450 (CYP) enzyme system, which plays a major role in breaking down corticosteroids. This induction accelerates steroid metabolism, reducing their effectiveness and posing serious clinical risks [11]. In our case, the patient did not have any signs of active TB infection; thus, he was kept on intravenous hydrocortisone (100 mg/m^2^/day) and was transitioned to maintenance oral hydrocortisone (15 mg/m^2^/day) and fludrocortisone (0.1 mg/day) only.

A pediatric case from Lima involved a 10‐year‐old boy who was diagnosed with primary adrenal insufficiency (PAI), where imaging revealed adrenal enlargement and calcification, findings that were later confirmed to be due to systemic tuberculosis [12]. In another case, a 38‐year‐old African male had adrenal tuberculosis successfully diagnosed through imaging and biopsy, despite the absence of pulmonary involvement [13]. These reports underscore the importance of recognizing imaging features such as adrenal enlargement or atrophy, combined with systemic symptoms, as strong indicators of adrenal TB, even when microbiological cultures are negative, similar to our case.

### 5.1 Screening and Long‐Term Management

In patients with prior tuberculosis who present with suggestive features (persistent hypotension, unexplained hypoglycemia, hyperpigmentation, or unexplained weight loss), targeted biochemical screening with a morning (08:00) serum cortisol ± ACTH and/or a standard ACTH stimulation test is reasonable. Long‐term management of confirmed primary adrenal insufficiency includes physiologic glucocorticoid replacement (e.g., hydrocortisone titrated to clinical response and growth in children), mineralocorticoid replacement with fludrocortisone when indicated, periodic monitoring of growth and electrolytes, patient/parent education on sick‐day rules, and provision of an emergency steroid card and injectable hydrocortisone for use during intercurrent illness or trauma.

## 6 Limitations

The limitations of this report include the lack of adrenal biopsy to definitively confirm TB as the cause of adrenal atrophy and limited long‐term follow‐up data.

## 7 Conclusion

This case emphasizes the importance of considering adrenal insufficiency in patients with a history of TB who present with nonspecific symptoms. Clinicians working in TB‐endemic areas should maintain a high index of suspicion for adrenal insufficiency in patients with suggestive clinical features (persistent hypotension, unexplained hypoglycemia, hyperpigmentation). Imaging, such as CT showing adrenal atrophy, can further support the diagnosis, as described in prior studies.

## Author Contributions

**Aisha Haleem:** conceptualization, data curation, formal analysis, writing – original draft, writing – review and editing. **Saad Ashraf:** conceptualization, project administration, supervision, writing – original draft, writing – review and editing. **Daniya Tarique:** conceptualization, writing – original draft, writing – review and editing. **Muhammad Zohair:** data curation, writing – review and editing. **Ajeet Singh:** validation, writing – original draft, writing – review and editing. **Biruk Demisse Ayalew:** supervision, writing – review and editing.

## Ethics Statement

This case report was exempt from formal ethical approval, as it involves a single patient with anonymized data; written informed consent was obtained from the parents for publication, including the use of clinical images.

## Conflicts of Interest

The authors declare no conflicts of interest.

## Data Availability

Clinical data from this case report is available upon reasonable request to the corresponding author and is subject to privacy restrictions.
